# Identification of Potent CD19 scFv for CAR T Cells through scFv Screening with NK/T-Cell Line

**DOI:** 10.3390/ijms21239163

**Published:** 2020-12-01

**Authors:** Chung Hyo Kang, Yeongrin Kim, Heung Kyoung Lee, So Myoung Lee, Hye Gwang Jeong, Sang Un Choi, Chi Hoon Park

**Affiliations:** 1 Therapeutics & Biotechnology Division, Korea Research Institute of Chemical Technology, P.O. Box 107, Daejeon 34114, Korea; kangch@krict.re.kr (C.H.K.); kyr3915@krict.re.kr (Y.K.); craet@krict.re.kr (H.K.L.); puresm@krict.re.kr (S.M.L.); suchoi@krict.re.kr (S.U.C.); 2 College of Pharmacy, Chungnam National University, Daejeon 34134, Korea; hgjeong@cnu.ac.kr; 3 Medicinal Chemistry and Pharmacology, Korea University of Science and Technology, Daejeon 34113, Korea

**Keywords:** CD19, chimeric-antigen receptor, scFv, FMC63, 4G7, leukemia

## Abstract

CD19 is the most promising target for developing chimeric-antigen receptor (CAR) T cells against B-cell leukemic cancer. Currently, two CAR-T-cell products, Kymriah and Yescarta, are approved for leukemia patients, and various anti-CD19 CAR T cells are undergoing clinical trial. Most of these anti-CD19 CAR T cells use FMC63 single-chain variable fragments (scFvs) for binding CD19 expressed on the cancer cell surface. In this study, we screened several known CD19 scFvs for developing anti-CD19 CAR T cells. We used the KHYG-1 NK/T-cell line for screening of CD19 scFvs because it has advantages in terms of cell culture and gene transduction compared to primary T cells. Using our CAR construct backbone, we made anti-CD19 CAR constructs which each had CD19 scFvs including FMC63, B43, 25C1, BLY3, 4G7, HD37, HB12a, and HB12b, then made each anti-CD19 CAR KHYG-1 cells. Interestingly, only FMC63 CAR KHYG-1 and 4G7 CAR KHYG-1 efficiently lysed CD19-positive cell lines. In addition, in Jurkat cell line, only these two CAR Jurkat cell lines secreted IL-2 when co-cultured with CD19-positive cell line, NALM-6. Based on these results, we made FMC63 CAR T cells and 4G7 CAR T cells from PBMC. In in vitro lysis assay, 4G7 CAR T cells lysed CD19-positive cell line as well as FMC63 CAR T cells. In in vivo assay with NOD.Cg-PrkdcscidIl2rgtm1Wjl/SzJ (NSG) mice, 4G7 CAR T cells eradicated NALM-6 as potently as FMC63 CAR T cells. Therefore, we anticipate that 4G7 CAR T cells will show as good a result as FMC63 CAR T cells for B-cell leukemia patients.

## 1. Introduction

T cells genetically modified to express chimeric-antigen receptor (CAR) specific to a tumor have shown dramatic results in preclinical and clinical tests [1]. Structurally, CAR is composed of an antigen-binding domain, which is typically a single-chain variable fragment (scFv) of monoclonal antibody (mAb), a costimulatory domain, and a CD3ζ domain of T-cell receptor (TCR) [2]. Clinical trials of CAR T cell-targeting CD19 have shown significant responses in acute lymphoblastic leukemia (ALL), chronic lymphocytic leukemia (CLL), diffuse large B-cell lymphoma (DLBCL), and follicular lymphoma (FL) etc. [3,4,5,6,7,8,9,10,11]. Two CD19 CAR T cell products are approved by the US FDA, and numerous clinical trials using CD19 CAR T cells are still ongoing by various institutions [12]. Each CD19 CAR T cell has a different CAR backbone, different gene transduction method, and different culture conditions [13,14]. For example, Kymriah, which is manufactured by Novartis, uses 4-1BB as the co-stimulatory domain, however, Yescarta, which is a Kite’s product, uses a CAR construct with CD28 as the co-stimulatory domain [15]. In addition, the National Cancer Institute (NCI) adopted γ-retrovirus for gene transduction in T cells, while the University of Pennsylvania (UPenn) adopted lentivirus for this purpose [16]. Although they have quite different strategies to manufacture CD19 CAR T cells, there is one thing which is common in most institutions—CD19 scFv. Nearly 90% of the worldwide clinical anti-CD19 CAR T cell products use FMC63 as a CD19 scFv [17]. However, so far, numerous CD19 scFvs targeting the CD19 extracellular domain have been invented [18,19]. Therefore we postulated that it is meaningful to test these CD19 scFvs for developing potent CD19 CAR T cells.

Here, we used the KHYG-1 NK/T cell line to test CAR construct with each CD19 scFv. It is not easy to make CAR T cells from peripheral blood mononuclear cells (PBMC) isolated from human blood. Because the KHYG-1 cell line has cytotoxic activity against target cells, KHYG-1 incorporated with CAR have the ability to lyse CAR-targeting cells [20,21,22]. Moreover, we proved that KHYG-1 is easy to culture it can easily transduce the CAR gene. Using the KHYG-1 cell line, we tested eight known CD19 scFvs for CAR T cells. Our in vitro and in vivo results revealed that 4G7 is as good as FMC63 for anti-CD19 CAR T cells.

## 2. Results

### 2.1. Usage of the KHYG-1 Cell Line to Screen CD19 scFvs for CD19 CAR T Cells

**Figure 1:**
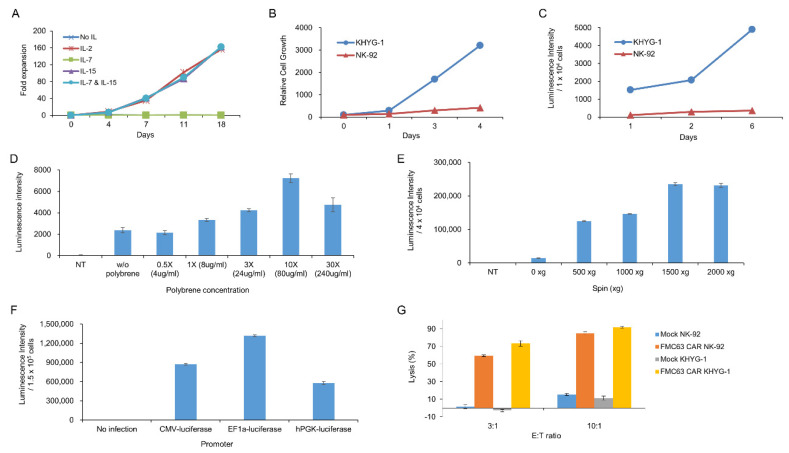
Biological characteristics of KHYG-1 cells used for CD19 single-chain variable fragment (scFv) screening. (**A**) The effect of cytokines, including IL-2, IL-7, and IL-15, on the proliferation of KHYG-1 cells was investigated. After adding IL-2, IL-7, IL-15, or IL-7/IL-15 to the culture medium, proliferation of KHYG-1 was analyzed by cell counting. (**B**) Comparison of cell proliferation in KHYG-1 and NK-92. After KHYG-1 and NK-92 were cultured in each culture medium containing IL-2, proliferation was measured by a cell titer-glo assay. (**C**) Comparison of transduction efficiency in KHYG-1 and NK-92. After luciferase transduction to KHYG-1 and NK-92, luminescence value was detected by bright-glo assay for 6 days. (**D**) The effect of polybrene on transduction efficiency in KHYG-1. After luciferase transduction with different concentrations of polybrene into KHYG-1, luminescence was measured using a bright-glo assay on the 4th day. (**E**) KHYG-1 cells were infected with luciferase containing lentiviral particles with different g-values. After 5 days, luminescence was measured. (**F**) Comparison of gene expression by promoter type in KHYG-1. KHYG-1 cells were transduced with pLVX-CMV-Luc, pLVX-EF1α-Luc, or pLVX-hPGK-Luc lentivirus. On day 4, intensity of luminescence was measured through a bright-glo assay. (**G**) Antigen-specific cytotoxicity of KHYG-1 and NK-92. Mock KHYG-1, FMC63 CAR KHYG-1, mock NK-92, or FMC63 CAR NK-92 cells (E:T ratio 3:1 or 10:1) were co-cultured with NALM-6 (1.5 × 10^4^ cells), a CD19 positive cell line expressing luciferase, for 5 h. Then, luminescence values were measured.

We chose the KHYG-1 cell line to screen CD19 scFvs. Making CAR T cells from PBMC is relatively difficult, expensive, and time-consuming. Therefore we needed an easy system to test CAR constructs instead of CAR T cells from PBMC. Here, we used the KHYG-1 cell line, which is an NK/T-cell line. By gene transduction using lentiviral particle followed by puromycin selection, we obtained the KHYG-1 cell line stably expressing CAR protein. We tested several cytokines for culturing KHYG-1. Figure 1A shows that IL2 or IL15, not IL-7, is indispensable for KHYG-1 growth. Although the NK-92 cell line is more popular than the KHYG-1 cell line among NK cell researchers, we found that KHYG-1 has several advantages over the NK-92 cell line. The NK-92 cell line needs a special medium, which is very expensive. However, we needed just RPMI1640 plus 10% FBS to culture KHYG-1. In addition, as shown in Figure 1B, KHYG-1 has a high proliferation rate compared to NK-92. The doubling time of KHYG-1 is just 24 h, however, it is more than 72 h in NK-92. Therefore, we can get enough KHYG-1 cells very quickly compared to NK-92 cells. Moreover, KHYG-1 is very easily transduced by lentivirus compared to NK-92. To compare the transduction efficiency of these two cell lines, we measured the luminescence after luciferase gene transduction using lentivirus into KHYG-1 and NK-92. As we can see in Figure 1C, the luminescence from KHYG-1 was much higher than NK-92. It means that we can easily transduce the CAR gene into KHYG-1 compared to NK-92. To optimize the best condition for lentiviral infection into KHYG-1, we tested the polybrene concentration (Figure 1D). Polybrene is known to enhance the lentiviral infection in human cell lines. Data showed that polybrene increased the infection efficiency slightly. Although 10X polybrene concentration enhanced the transduction efficiency 3.5 fold, this high concentration of polybrene is very toxic to KHYG-1. The effect of polybrene on lentiviral infection into KHYG-1 seems to be very weak compared to other cancer cell lines. We checked whether transduction efficiency in KHYG-1 is enhanced by spinoculation. Spinoculation increased gene transduction greatly by more than 10-fold (Figure 1E) and 1500× *g* seemed to be best for KHYG-1 spinoculation. We tested which promoter is best for gene expression in the KHYG-1 cell line. We infected cytomegalovirus (CMV), elongation factor-1 alpha (EF1α), or phosphoglycerate kinase (PGK) promoter containing the luciferase gene into KHYG-1 using lentivirus. Figure 1F shows that EF-1a promoter was superior to other promoters. Finally, we compared the lytic activity of CAR NK-92 and CAR KHYG-1 against tumor cells. Both FMC63 CAR NK-92 and FMC63 CAR KHYG-1 efficiently eliminated CD19-positive cell line, NALM-6 (Figure 1G). Lytic activity of FMC63 CAR KHYG-1 seemed to be slightly better than FMC63 CAR NK-92. Neither mock NK-92 nor mock KHYG-1 lysed the CD19 target cells. These data means that both cell lines are able to perform target-specific lysis.

### 2.2. FMC63 and 4G7 scFvs Are Proved to Be Applicable for Anti-CD19 CAR Construct

**Figure 2:**
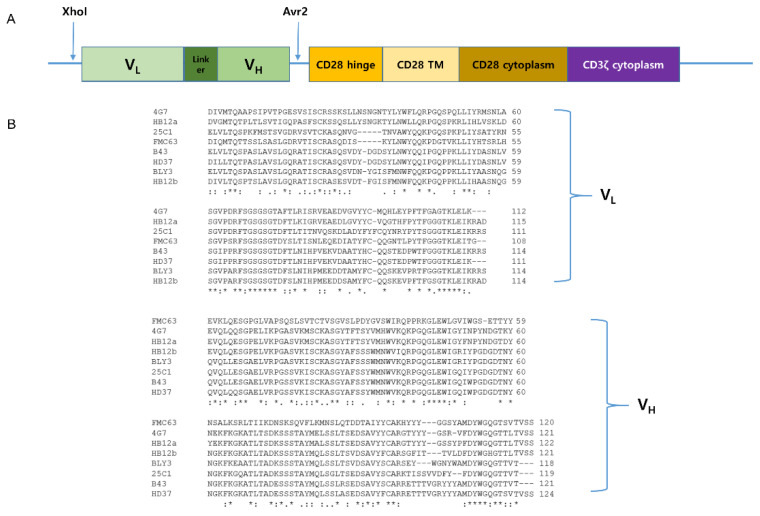
CAR constructs of various scFvs capable of targeting CD19. (**A**) Schematic representation of anti-CD19 CAR construct. It is a second-generation CAR composed of anti-CD19 scFv, CD28 transmembrane region, CD28 co-stimulatory domain, and CD3ζ signaling domain. (**B**) V_H_ and V_L_ sequences of eight different anti-CD19 scFvs.

**Figure 3:**
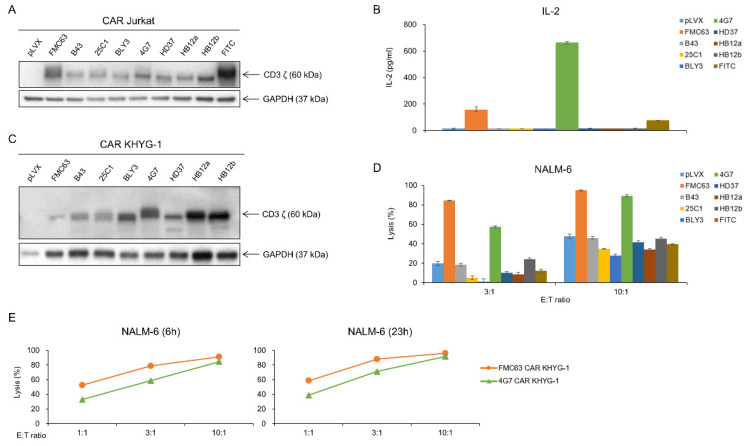
FMC63 and 4G7 CAR constructs effectively recognize antigen CD19. (**A**) After transfection of CD19 CAR genes into Jurkat cells, CAR expression was confirmed through the Western blot. (**B**) CD19 CAR Jurkat (2 × 10^5^ cells) co-cultured with 2 × 10^4^ NALM-6 cells for 21 h. IL-2 level secreted by CD19 CAR Jurkat was measured through ELISA assay. (**C**) After transduction of eight different CD19 CAR genes into KHYG-1 cells, CAR expression was confirmed through Western blot. (**D**) Tumor-killing ability of eight different CD19 CAR KHYG-1 cells against luciferase-expressing NALM-6. CD19 CAR KHYG-1 cells (E:T ratio 3:1 or 10:1) were co-cultured with luciferase-expressing NALM-6 (1.5 × 10^4^ cells) for 7 h. Then, luminescence values were measured. (**E**) FMC63 CAR KHYG-1 or 4G7 CAR KHYG-1 cells were incubated with NALM-6 at different E:T ratios (1:1, 3:1, and 10:1) and different incubation times (6 h and 23 h) to see their lytic activity.

Our lab has the CAR construct backbone. In this construct, we can change any scFvs using XhoI and AvrII restriction enzyme by cloning. We made eight different anti-CD19 CAR plasmids, which each had a CD19 scFv, including FMC63, B43, 25C1, BLY3, 4G7, HD37, HB12a, and HB12b. V_H_ and V_L_ sequences of each scFv are shown in Figure 2. We checked the availability of CD19 scFvs for CAR T cells using the Jurkat cell line. Each anti-CD19 CAR construct was transiently transfected into Jurkat cells (Figure 3A). CAR Jurkat cells were incubated with NALM-6, a CD19-positive cell line. Only FMC63 and 4G7 CAR Jurkat cells secreted IL-2 (Figure 3B). To compare the lytic activities of each CD19 CAR construct, we made lentivirus for CAR transduction into KHYG-1 cell by spinoculation. After puromycin selection for 2 weeks, we confirmed the CAR expression in each KHYG-1. As shown in Figure 3C, each CAR protein was expressed in KHYG-1. Using these CD19 CAR KHYG-1 cells, we performed lysis assay with NALM-6. FMC63 and 4G7 CAR KHYG-1 lysed NALM-6 very efficiently. Interestingly, anti-CD19 KHYG-1 cell lines equipped with other CD19 scFvs, including B43, 25C1, BLY3, HD37, HB12a, and HB12b, did not lyse NALM-6 (Figure 3D). Figure 3E shows that the in vitro lytic activity of FMC63 CAR KHYG-1 was slightly higher than 4G7 CAR KHYG-1. These data mean that FMC63 and 4G7 CAR KHYG-1 cells are able to trigger cell lysis of CD19 positive cells.

### 2.3. 4G7 CAR T Cells Are as Effective as FMC63 CAR T Cells in Eradicating Leukemic Tumor Cells

**Figure 4:**
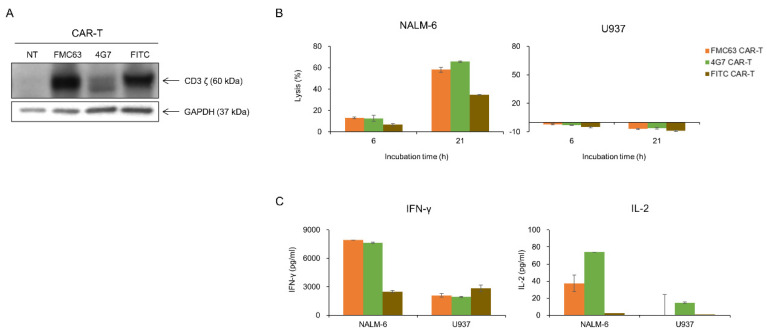
FMC63 and 4G7 CAR T cells effectively responded to CD19-positive NALM-6 cells. (**A**) After transduction of anti-CD19 CAR lentiviruses into primary human T cells, CAR expression was confirmed through the Western blot. Anti-human CD3 antibody was used to detect CD3ζ of CAR. (**B**) Tumor-killing ability of FMC63, 4G7, and FITC CAR T cells against luciferase-expressing NALM-6 and U937. FMC63, 4G7, and FITC CAR T cells (2 × 10^5^ cells) were co-cultured with luciferase-expressing NALM-6 (2 × 10^4^ cells) or U937 (2 × 10^4^ cells) for indicated incubation times at the E:T ratio 10:1. Then, luminescence values were measured. (**C**) FMC63, 4G7, or FITC CAR T cells (1.5 × 10^5^ cells) were co-cultured with NALM-6 cells (1.5 × 10^4^ cells) or U937 cells (1.5 × 10^4^ cells) for 21 h at the E:T ratio 10:1. The amount of IFN-γ and IL-2 secreted by CAR T cells was measured through the ELISA assay.

**Figure 5:**
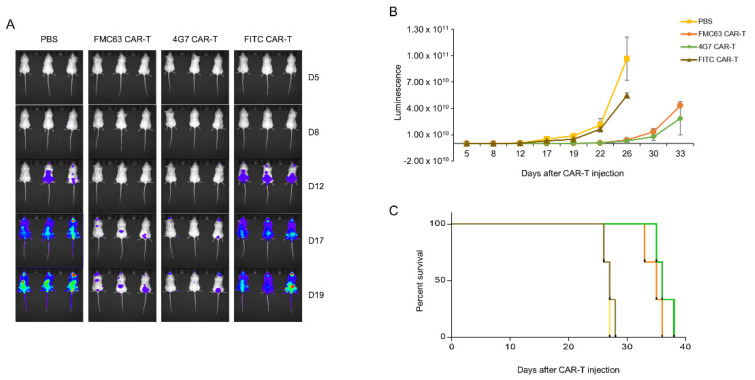
In vivo, both FMC63 and 4G7 CAR T cells eliminated acute-lymphoblastic-leukemia-cell NALM-6. (**A**–**C**) NOD.Cg-PrkdcscidIl2rgtm1Wjl/SzJ (NSG) mice were injected intravenously (i.v.) with 5 × 10^6^ Luc-expressing NALM-6 cells. Next day, mice were administered with 1 × 10^7^ FMC63, 4G7, and FITC CAR T cells or phosphate buffered saline (PBS) control (i.v.). From day 5, tumor progression was observed via bioluminescence imaging. (**A**) Represents the luminescence image, and (**B**) indicates the luminescence value of each group. (**C**) Percentage survival was calculated.

As 4G7 had proven to be as good as FMC63 in lysing NALM-6 in KHYG-1 cell model, we determined to make anti-CD19 CAR T cells with 4G7 scFv to compare with FMC63. We made 4G7 and FMC63 CAR T cells with PBMC isolated from healthy donor’s blood. As shown in Figure 4A, CAR expressions were confirmed in both CAR T cells. Both CAR T cells selectively lysed the CD19-positive cell line (Figure 4B). U937, a CD19 negative cell line, was not lysed by CD19 CAR T cells. Also, IFN-γ and IL-2 were secreted when FMC63 and 4G7 CAR T cells were incubated with NALM-6, not with U937 (Figure 4C). We performed in vivo assay with an NSG mice model. NALM-6 cells expressing luciferase were injected into NSG mice by i.v. route. After 1 day, CAR T cells were injected by i.v. route. In the in vivo model, both FMC63 and 4G7 CAR T cells eradicated NALM-6, while control CAR T cells (FITC CAR T) did not (Figure 5A,B). The survival curve also shows similar efficacy between 4G7 CAR T and FMC63 CAR T cells (Figure 5C). These data suggest that 4G7 CAR T cells are as effective as FMC63 CAR T cells against B-cell leukemia.

## 3. Discussion

Because of the high response rate of anti-CD19 CAR T cells in leukemia patients, numerous anti-CD19 CAR T cell clinical trials are in process worldwide [23,24,25,26,27]. Although there are many qualified CD19 antibodies, most of clinical trials of CD19 CAR T cells use FMC63 exclusively [13,28]. Recently, several institutes have been trying to use novel CD19 scFvs, which they developed, for clinical trial of CAR T cell therapy [29,30,31]. We searched for CD19 scFvs in literature including patents and scientific papers. We found that more CD19 scFvs that bind CD19 effectively exist. We tried to make CAR constructs with these CD19 scFvs, and finally we got eight CD19 CAR constructs as shown in Figure 2. Here, we tested eight known CD19 antibodies, FMC63, 4G7, 25C1, B43, HD37, BLY3, HB12a, and HB12b, for CD19 CAR T cells. These antibodies are old, because, except for HB12a and HB12b which were developed in 2011, these CD19 scFvs were developed in the 1980s or 1990s. They were also produced in different ways. FMC63 is produced by immunizing BALB/c mice with JVM3, a B-CLL cell line [19]. 4G7 was derived by immunizing mice with B-CLL cells. HD37 was produced by immunizing BALB/c mice with hairy cell leukemia cells [32]. B43 was derived by immunizing BALB/c mice with Burkitt’s lymphoma cells [33]. These CD19 scFvs were cloned in our CAR construct comprised of CD28 and CD3ζ cytoplasmic domains. We used KHYG-1 cell to screen CD19 scFvs. Because the KHYG-1 cell line is very easy to culture, we selected KHYG-1 for our study instead of NK-92, which is a very popular cell line in CAR T cell research as it can grow on very simple medium (RPMI1640 supplemented with 10% FBS), and it grows very fast (Figure 1B). On the contrary, NK-92 needs special medium, which is very expensive, and it grows very slowly. In addition, we can more easily transduce CAR gene into KHYG-1 cell line than the NK-92 cell line (Figure 1C). Most researchers use the NK-92 cell line for CAR T-cell study [34,35,36,37,38,39]. However, several research groups use KHYG-1 cell line for CAR T-cell study [20,21,22]. Using this system, we made eight different anti-CD19 CAR KHYG-1 cells, which had different CD19 scFvs to each other. CAR KHYG-1 data show that only FMC63 and 4G7 CAR KHYG-1 lyse NALM-6 cells very efficiently in vitro. The Jurkat system also shows that FMC63 and 4G7 CAR Jurkat cells secrete IL-2 when incubated with CD19-positive cell lines. Based on these data, we made CD19 CAR T cells with FMC63 or 4G7 scFvs from peripheral blood mononuclear cells (PBMC). These two CAR T cells lysed CD19-positive cell lines, and secreted IFN-γ and IL-2 when incubated with CD19-positive cell lines. As shown in Figure 5, the anti-tumor efficacy of 4G7 CAR T cells is as good as FMC63 CAR T cells. Although our data strongly suggest that 4G7 CAR T cells are very powerful against CD19-positive tumors, there is no clinical trial of CD19 CAR T cells using 4G7 scFv. Based upon our data, we postulate that the therapy with 4G7 CAR T cells against B-cell lymphoma would be as successful as therapy with FMC63 CAR T cells. Our data show that only FMC63 and 4G7 are effective for CD19 CAR T cells. Currently, we do not know exactly why only these two scFvs are effective in CD19 CAR T cells. One reason could be that spacer and transmembrane domain might cause changes in 3-dimensional structure in scFvs to the binding affinity to the epitope. In further study, we can try to make a CD19 CAR construct that has a different spacer or transmembrane domain to increase the activity of CAR T cells.

Here we suggest that KHYG-1 is very useful cell line for CAR T-cell study. Also, we suggest the 4G7 scFv can be a potent CD19 scFv for making CD19 CAR T cells. In vitro and in vivo data demonstrate that 4G7 CAR T cells perform anti-cancer activity as effective as FMC63 CAR T cells. Since the current FMC63 CAR T cells show excellent efficacy in patients, the development of new CD19 CAR T cells should be focused on FMC63 CAR-T-cell-resistant patients. Although FMC63 and 4G7 recognize a similar conformational epitope centered on residue R144 in CD19, deep mutational scanning experiments demonstrated that the residues of CD19 which affect the binding to CD19 are slightly different between FMC63 and 4G7 [40,41,42,43]. Therefore, it is plausible that the 4G7 CAR T cell can be used for FMC63 CAR-T-cell resistant patients. In addition, recent papers demonstrate that the transmembrane or spacer domain of CAR influences the function of CAR T cell [44]. Therefore, in further study, we can try making 4G7 CAR T cells with optimized transmembrane or spacer domains to increase the anti-tumor effect.

## 4. Materials and Methods

### 4.1. Cell Culture

NALM-6 and Jurkat cells were cultured in RPMI1640 medium (SH30027; HyClone Laboratory Tools, Marlborough, MA, USA) supplemented with 10% fetal bovine serum (FBS) (16000-044; Gibco Life Technologies, Carlsbad, CA, USA). KHYG-1 and T cells were maintained in RPMI1640 supplemented with 10% FBS and 200 IU/mL rhIL-2 (202-IL-500; R&D Systems, Minneapolis, MN, USA). NK-92 cells were cultured with Myelocult^TM^ H5100 (05150; STEMCELL Technologies, Vancouver, Canada) with 500 IU/mL rhIL-2. Cells were incubated in a humidified incubator at 37 °C with 5% CO_2_.

### 4.2. Construct of CARs

Eight CD19 scFvs (FMC63, B43, 25C1, BLY3, 4G7, HD37, HB12a, and HB12b) and FITC and scFvs were synthesized by Macrogene (Daejeon, Korea). scFvs recognize the fluorescein derivative called fluorescein isothiocyanate (FITC). Since FITC did not exist in the cells, CAR-targeting FITC was used as a negative control. Eight CD19 CAR and FITC CAR genes were inserted into the pLVX-CMV-puro (632164; Clontech, Mountain View, CA, USA) vector or into pLVX-EF1α vector (631982; Clontech) in which IRES-ZsGreen was deleted.

### 4.3. Lentivirus Production and Generation of CAR KHYG-1

On day 0, 1.5 × 10^7^ 293T cells were seeded in a 150-mm dish. Next day, eight different CD19 CAR, FITC CAR, and luciferase vectors were co-transfected with lentiviral packaging plasmids, pRSV-Rev (12253; Addgene, Watertown, MA, USA), pMDLg/pRRE (12251; Addgene) and pMD2.G (12259; Addgene) to 293T cells. On day 2, the medium of transfected 293T cells was replaced with fresh medium. On day 3–4, lentivirus supernatants were collected from the transfected 293T cells and were filtered with 0.45 μm a polyethersulfone (PES) membrane filter (SLHP033RB; Merck Millipore, Burlington, MA, USA). If necessary, lentivirus supernatants were concentrated using Lenti-X concentrator (631232; Clontech).

### 4.4. Human T Cell Isolation and Generation of CAR T Cells

Whole blood was obtained from the Korean Red Cross Blood Services and peripheral blood mononuclear cells (PBMCs) were separated using density-gradient centrifugation. Whole blood was diluted with an equal volume of PBS containing 2% FBS. Fifteen mL of Lymphoprep (07851; STEMCELL Technologies) was added to the conical centrifuge tube, then 30 mL of diluted whole blood was added. Samples were centrifuged at 800× *g* for 20 min without a break and the yellow middle layer (mononuclear cells, MNCs) was transferred to a new conical centrifuge tube. After adding the wash buffer (PBS with 2% FBS) to MNCs, tubes were centrifuged at 300× *g* for 8 min. This was repeated twice.

On day 0, PBMC were cultured in culture medium containing Dynabeads Human T-Activator CD3/CD28 (11132D; Thermo Fisher Scientific, Waltham, MA, USA) and 200 IU/mL rhIL-2 to activate only T cells. On the day 2, the Dynabeads were removed with an EasySep Magnet (18001; STEMCELL Technologies) and then mixed with lentivirus supernatants. Eight μg/mL of polybrene was added to the T cell-lentivirus mixture and spinoculation was done for 90 min at 1000× *g*. After centrifugation, transduced T cells were incubated in a medium containing rhIL-2. On day 7, to evaluate the function of CAR T cells, cytotoxicity assay and cytokine release assay were performed, as well as Western blot to confirm CAR expression.

### 4.5. Proliferation Assay

To compare the effect of interleukins on proliferation of KHYG-1 cells, 1 × 10^5^ KHYG-1 cells were cultured in medium containing IL-2 (202-IL-500; R&D Systems), IL-7 (200-07; PeproTech, Cranbury, NJ, USA), IL-15 (200-15; PeproTech), or IL-7/IL-15, respectively. On days 1, 2, and 4, the number of KHYG-1 cells cultured in each medium were counted using a TC20 Automated Cell Counter (Bio-Rad Laboratories, Hercules, CA, USA).

To compare the proliferation of KHYG-1 and NK-92 cells, 2 × 10^3^ NK-92 and KHYG-1 cells were seeded in a 96-well plate and incubated at 37 °C. On days 1, 3, and 4, equal volumes of CellTiter-Glo assay reagent (G7572; Promega, Madison, WI, USA) was added to each well and shaken for 2 min. To stabilize the luminescence signal, the plate was left at room temperature for 10 min, and then the luminescence was measured via an EnVision reader (PerkinElmer, Waltham, MA, USA).

### 4.6. Cytotoxicity Assay

To generate target cells expressing luciferase, lentiviral luciferase was transduced into NALM-6. In 96-well plates, CAR NK-92, CAR KHYG-1, or CAR T cells were co-cultured with luciferase-expressing NALM-6 at indicated E:T ratio and incubation time. At the end of incubation time, the Bright-Glo Luciferase assay reagent (E2650; Promega) was added to each well containing effector/target cell mixture. After shaking the plate for 5 min at room temperature, luminescence was detected using an EnVision reader (PerkinElmer).

### 4.7. Cytokine Release Assay

Eight CD19 CAR (FMC63, B43, 25C1, BLY3, 4G7, HD37, HB12a, and HB12b) and FITC CAR genes were transduced into Jurkat or T cells by electroporation or lentiviral infection, respectively. CAR Jurkat and CAR T cells were co-cultured with NALM-6 for the indicated E:T ratio and indicated incubation time in 96-well plates. At the end of incubation, effector–target cell mixtures were harvested in 1.5 mL microtubes and centrifuged at 4 °C, 13,000 rpm for 10 min. Only supernatants were collected and cytokine level was measured using human interleukin-2 (IL-2, 431801; BioLegend, San Diego, CA, USA) and human interferon gamma (IFN-γ, 430104; BioLegend) ELISA assay kit.

### 4.8. Western Blotting

CAR transduced Jurkat, KHYG-1, and T cells were harvested and cell lysates were made by 1× sample buffer (10% glycerol, 2% SDS, 50 mM Tris-HCl (pH 6.8), 3% β-mercaptoethanol). Lysates were boiled 10 min, at 95 °C. Samples were loaded on 4–15% gradient Mini-PROTEAN TGX gels (456-1086; Bio-Rad Laboratories) and electrophoresis was performed at 100 V for 75 min. The transfer step was conducted at 250 mA for 60 min. Using the blocking buffer (5% skimmed milk in 1× TBS-T), membrane was blocked for 60 min. After washing with 1X TBS-T, the membrane was incubated with primary antibody (CD3ζ (551034; BD Biosciences, San Jose, CA, USA), GAPDH (5174; Cell Signaling Technology, Danvers, MA, USA)) overnight at 4 °C. Next day, the membrane was washed with 1× TBS-T and incubated with a horseradish peroxidase (HRP)-conjugated secondary antibody (31430 and 31460; Thermo Fisher Scientific). Signal intensity was detected using SuperSignal West Pico solution (1863096 and 1863097; Thermo Fisher Scientific) and Sensi-Q2000 Chemidoc (LugenSci, Bucheon, Korea) instruments.

### 4.9. Mouse Xenograft Model

The NSG (NOD.Cg-PrkdcscidIl2rgtm1Wjl/SzJ) mice were purchased from Charles River Laboratories (Wilmington, MA, USA) and the experimental procedure was conducted according to guidelines approved by the Laboratory Animal Care and Use Committee of the Korea Research Institute of Chemical Technology (project code: 2020-6C-01-01, approval date: 20 January 2020). The NSG mice were injected intravenously (i.v.) with 4 × 10^5^ luciferase-expressing NALM-6 cells. On day 1, control (PBS), FMC63 CAR T, 4G7 CAR T, or FITC CAR T (1 × 10^7^) cells were administered by i.v. to NSG mice. To measure tumor progression, d-luciferin (122799; PerkinElmer) was administered intraperitoneally to mice, and luminescence was monitored using the IVIS Spectrum In vivo Imaging System (PerkinElmer).

## Abbreviations

ALL	Acute lymphoblastic leukemia
CAR	Chimeric antigen receptor
CLL	Chronic lymphocytic leukemia
CMV	Cytomegalovirus
DLBCL	Diffuse large B-cell lymphoma
EF1α	Elongation factor-1 alpha
FL	Follicular lymphoma
HRP	Horseradish peroxidase
IFN-γ	Interferon-gamma
IL-2	Interleukin-2
mAb	Monoclonal antibody
MNC	Mononuclear cells
NCI	National Cancer Institute
NK	Natural killer
NSG	NOD.Cg-PrkdcscidIl2rgtm1Wjl/SzJ
PBMC	Peripheral blood mononuclear cell
PBS	Phosphate buffered saline
PES	Polyethersulfone
PGK	Phosphoglycerate kinase
scFv	Single-chain variable fragment
TCR	T-cell receptor
UPenn	University of Pennsylvania
